# Antibacterial activity of endophytic fungi from leaves of *Indigofera suffruticosa* Miller (Fabaceae)

**DOI:** 10.3389/fmicb.2015.00350

**Published:** 2015-05-07

**Authors:** Irailton Prazeres dos Santos, Luís Cláudio Nascimento da Silva, Márcia Vanusa da Silva, Janete Magali de Araújo, Marilene da Silva Cavalcanti, Vera Lucia de Menezes Lima

**Affiliations:** ^1^Departamento de Micologia, Centro de Ciências Biológicas, Universidade Federal de PernambucoRecife, Brazil; ^2^Departamento de Bioquímica, Centro de Ciências Biológicas, Universidade Federal de PernambucoRecife, Brazil; ^3^Departamento de Antibióticos, Centro de Ciências Biológicas, Universidade Federal de PernambucoRecife, Brazil

**Keywords:** antibacterial activity, endophytic microorganisms, minimum inhibitory concentration, *Nigrospora sphaerica*, *Staphylococcus aureus*

## Abstract

Endophytic fungi were isolated from healthy leaves of *Indigofera suffruticosa* Miller, a medicinal plant found in Brazil which is used in folk medicine to treat various diseases. Among 65 endophytic fungi isolated, 18 fungi showed activity against at least one tested microorganism in preliminary screening, and the best results were obtained with *Nigrospora sphaerica* (URM-6060) and *Pestalotiopsis maculans* (URM-6061). After fermentation in liquid media and in semisolid media, only *N. sphaerica* demonstrated antibacterial activity (in Potato Dextrose Broth-PDB and in semisolid rice culture medium). In the next step, a methanolic extract from rice culture medium (NsME) and an ethyl acetate extract (NsEAE) from the supernatant of PDB were prepared and both exhibited antimicrobial activity against Gram-negative and Gram-positive bacteria. The best result was observed against *Staphylococcus aureus*, with minimum inhibitory concentration (MIC) and minimum bactericidal concentration (MBC) values of 1.56 mg/mL and 6.25 mg/mL, respectively, for NsME and MIC and MBC values of 0.39 mg/mL and 3.12 mg/mL, respectively, for NsEAE. This study is the first report about the antimicrobial activity of endophytic fungi residing in *I. suffruticosa* leaves, in which the fungus *N. sphaerica* demonstrated the ability to produce bioactive agents with pharmaceutical potential, and may provide a new lead in the pursuit of new biological sources of drug candidates.

## Introduction

The development of resistance by existing pathogenic bacteria and fungi to commercial drugs is a relevant problem faced by health services (Costelloe et al., 2010) and has become a serious concern around the world (Aksoy and Unal, 2008). Several factors have favored this scenario, such as extensive and often inappropriate use of antibiotics, poor hygienic conditions, continuous movement of travelers, increased numbers of immunocompromised patients, and delay in diagnosis of infections (von Nussbaum et al., 2006). As a result, an intensive search for new, effective antimicrobial agents is necessary, which is facilitated by exploring new niches and habitats (Xing et al., 2011; Zhao et al., 2011a).

A range of microbial species are known to be endophytic, colonizing inter- and intracellular spaces of plant tissues without causing apparent damage and appearing to be associated with all plants in natural ecosystems (Rodriguez et al., 2009). Among endophytic microorganisms, fungi have an intimate relationship with host plants and can produce compounds that promote vegetative growth, competitiveness and protection of the host against herbivores and pathogens (Porras-Alfaro and Bayman, 2011). Endophytic fungi represent a wide diversity of microbial adaptations that have evolved in special and unusual environments, making them a great source of study and research for new drugs for medical, industrial, and agriculture uses (Yu et al., 2010; Li et al., 2012; Teiten et al., 2013; Mapperson et al., 2014). These microorganisms are well known to produce bioactive secondary metabolites such as alkaloids, terpenoids, steroids, quinones, isocoumarins, lignans, phenylpropanoids, phenols, and lactones (Radić and Štrukelj, 2012; Deshmukh et al., 2014).

Plants used in traditional medicine have played a very important role in the search for new bioactive strains of endophytic fungi, as it is possible that their beneficial characteristics are a result of the metabolites produced by their endophytic community (Kaul et al., 2012; Kusari et al., 2013). Despite this potential, a repertoire of medicinal plants remains to be studied regarding their endophytic composition, for example *Indigofera suffruticosa* Miller. This is a well-known Brazilian medicinal plant whose leaves have been proven to have anti-inflammatory, anticonvulsant antimicrobial, and wound-healing properties (Leite et al., 2004, 2006; Carli et al., 2010; Luiz-Ferreira et al., 2011; Almeida et al., 2013; Chen et al., 2013; Bezerra dos Santos et al., 2015). Due to the medicinal properties of *I. suffruticosa*, this species was the focus in the present study of a search for endophytic fungi able to produce bioactive substances with antimicrobial activity.

## Materials and Methods

### Plant Samples

The collection of plant samples was performed in Igarassu (07°50′00^′′^S 34°54′30^′′^W), Atlantic Coastal Forest, Pernambuco, Brazil. Samples were immediately processed in the Mycology Department, Federal University of Pernambuco (UFPE), Recife, Brazil.

### Isolation of Endophytic Fungi

In order to eliminate epiphytic microorganisms, plant samples were superficially sterilized by the method described by Araújo et al. (2002). Healthy leaves of *I. suffruticosa* were washed in running tap water, followed by immersion in 70% ethanol for 1 min, in sodium hypochlorite (2.0–2.5% available chlorine) for 4 min, in 70% ethanol for 30 s and washed three times with sterilized, distilled water. The efficiency of sterilization was confirmed by inoculating water from the last washing into Petri dishes containing potato dextrose agar (PDA; containing potato (200 g/L), dextrose (20 g/L), and agar (15 g/L), pH 6.0). After surface sterilization, the samples were cut into 0.5 cm^2^ pieces and aseptically transferred to Petri dishes containing PDA culture medium supplemented with chloramphenicol (100 μg/mL) to suppress bacterial growth. The Petri dishes were incubated at 30°C for 30 days and checked daily, and all fungal colonies found were isolated, purified and maintained in PDA for later testing.

### Screening of Antimicrobial Acitivity

#### Tested Microorganisms

Among the microorganisms used for antimicrobial tests, five were fungi pathogenic to humans (*Candida albicans,* URM-5852; *Epidermophyton flocosum,* URM-5510; *Malassezia furfur,* URM-5389; *Microsporum gypsseum,* URM-5478; and *Trichophyton mentagrophytes,* URM-5589) URM Culture Collection (WDCM604) of the Federal University of Pernambuco, Recife, Brazil. The other five microorganisms were bacteria obtained from the Culture Collection UFPEDA, Department of Antibiotics, UFPE, Recife, Brazil, two of which were Gram-positive bacteria (*Staphylococcus aureus*, UFPEDA-02; and *Bacillus subtilis*, UFPEDA-16), and three were Gram-negative (*Escherichia coli*, UFPEDA-224; *Klebsiella pneumoniae*, UFPEDA-396; and *Pseudomonas aeruginosa*, UFPEDA-39).

#### Antimicrobial Assay

The endophytic fungi were subjected to an antimicrobial assay using a solid medium (Ichikawa et al., 1971) which permits a rapid and qualitative selection of the bioactive microorganisms. Each endophytic strain was cultivated on the surface of PDA in Petri dishes, at 30°C, for 7 days. Then, disks were cut from the PDA plate (6 mm diameter) and transferred to the surface of Petri dishes previously spread with bacteria (Müller-Hinton agar, MHA) and fungi [Sabouraud dextrose agar (SDA) and SDA supplemented with 0.5% olive oil for *M. furfur*]. The Petri dishes were incubated at 37°C during 24 h for bacterial growth, and at 30°C during 48 h for fungal growth. Antimicrobial activity was assayed by the measurement of any inhibition diameter zones (IDZ).

### Identification of Endophytic Fungi

The identification of endophytic fungi was performed at the Mycology Department, UFPE, Recife, Pernambuco, Brazil, by means of analysis of macroscopic and microscopic characteristics of colonies. After identification, fungi that showed antimicrobial activity were stored in the URM Culture Collection (WDCM604) of the Federal University of Pernambuco, Recife, Brazil.

### Fermentation Assay in Liquid Medium

Strains that showed the best results in the antimicrobial screening were further examined using a diffusion assay, in order to provide a way to select the best medium and incubation time for the production of the bioactive metabolites. Pre-inoculum were prepared in 250 mL Erlenmeyer flasks by adding five plugs (6 mm of diameter) of growing culture and 50 mL of PDB (potato: 200 g/L; dextrose: 20 g/L; pH 6.0). All cultures were incubated at 28 ± 2°C, on a rotary shaker, at 180 rpm. An aliquot (10 mL) of each pre-inoculum was transferred to 500 mL Erlenmeyer flasks containing 90 mL of the following media: PDB, Sabouraud broth (SAB), Malt Extract Broth (MEB; malt extract: 20 g/L), or Eurimycin Production Medium (EPM; soy flour: 20 g/L; glucose: 20 g/L; CaCO_3_: 2 g/L; and NaCl: 5 g/L). The flasks were incubated under the same conditions (28 ± 2°C, on a rotary shaker, at 180 rpm for 96 h). Every 24 h, samples of 1 mL from the fermentation broth were centrifuged for 15 min (Siqueira et al., 2011). For the fermentation in the liquid medium PDB, the endophytic fungi were grown on PDA, at 25°C, for 5 days. Three pieces (0.5 cm × 0.5 cm) of mycelial agar plugs were inoculated into 500 mL Erlenmeyer flasks containing 300 mL of potato dextrose broth and incubated at room temperature for 4 weeks. The cultures were filtered and the wet mycelia were discarded (Trisuwan et al., 2008). For the antimicrobial activity test, 30 μL of each supernatant obtained was utilized according to the disk diffusion method proposed by Bauer et al. (1966).

### Fermentation Assay in Semisolid Medium

The endophytes that showed the largest IDZ against the largest number of test microorganisms were cultured in the center of Petri dishes containing the medium PDA at 28 ± 2°C for 7 days, and from these colonies, five blocks of 5 mm diameter were transferred to Erlenmeyer flasks (1000 mL) containing the rice or corn semisolid media.

The preparation of semi-solid media was performed according to the methodology described by Aly et al. (2008), where 100 g of commercially available rice or corn and 100 mL of distilled water were added to Erlenmeyer flasks, autoclaved three times on alternate days and cultivated with the fungi *N. sphaerica* and *P. maculans* in static conditions at room temperature for 30 days. After the incubation period, methanol (300 mL) was added to each Erlenmeyer flask, followed by maceration. After 24 h, each sample was subjected to gravity filtration. The filtrate was concentrated on a rotary evaporator under reduced pressure at 50°C to obtain the methanolic extracts (NsME). The extract was kept at -20°C, and dissolved in dimethyl sulfoxide (DMSO) when ready for use.

### Preparation of Ethyl Acetate Extract From Fermentation Assay in Liquid Medium (PDB)

The method described by Trisuwan et al. (2008) was used, where after the fourth week, the culture fermented by *N. sphaerica* was filtered in vacuum filter using a no.3 Buchner funnel. The culture filtrates were extracted with ethyl acetate (2 × 300 mL) by partitioning in a separating funnel (solvent–solvent extraction). The culture filtrates (ethyl acetate extract –NsEAE) was concentrated on a rotary evaporator under reduced pressure at 50°C. The extract was kept at -20°C, and dissolved in dimethyl sulfoxide (DMSO) when ready for use.

### Phytochemical Analysis

Phytochemical analytical tests were performed to detect the presence of steroids, saponins, alkaloids, flavonoids, tannins, reducing compounds, terpenoids, cinnamic derivatives, and anthracene derivatives, according to the method described by Kokate (1994) and Harborne (1998).

### Determination of Minimum Inhibitory and Minimum Bactericidal Concentrations

A broth microdilution susceptibility assay was used for the determination of minimum inhibitory concentration (MIC) and minimum bactericidal concentration (MBC), as recommended by the National Committee for Clinical Laboratory (National Committee for Clinical Laboratory Standards [NCCLS], 2009). All tests were performed in Muller-Hinton broth. Bacteria were cultured overnight at 37°C. The test samples of the extracts were dissolved in 10% DMSO. Dilutions were prepared in 96-well microtiter plates to get final concentrations ranging from 0 to 50 mg/mL. After this step, each well received 10 μL of the suspension of microorganisms and 100 μL of liquid culture media. Plates were incubated at 37°C for 24 h; 15 μL of 0.01% resazurin was added as a colorimetric indicator of oxide reduction to characterize cell viability. Then the microplates were re-incubated for 4 h, and the lowest concentration of the extract that inhibited microbial growth was recorded as the MIC. Using the results of the MIC assay, the concentrations showing a complete absence of visual growth of bacteria were identified, and 50 μL of each culture broth were transferred onto the agar plates and incubated for the specified time and temperature as mentioned above. The complete absence of growth on the agar surface at the lowest sample concentration was defined as the MBC. Each assay in this experiment was replicated three times.

### Statistical Analysis

Data were analyzed using GraphPad Prism by one-way analysis of variance (ANOVA) and Tukey to determine statistical significance. A *p*-value of < 0.05 was considered to be statistically significant. The correlation index was calculated using the Pearson coefficient (ρ).

## Results

### Antimicrobial Activity Screening of Endophytic Fungi From Leaves of *I. suffruticosa*

A total of 65 endophytic strains were isolated from leaves of *I. suffruticosa* and subsequently submitted to a preliminary antimicrobial screening on solid medium. A total of 18 endophytic isolates showed activity against at least two of the tested bacteria, thus 33.6% of the isolates were found to be active and the majority of these endophytic strains (except for *N. sphaerica* ISEF 13) also showed a wide spectrum, inhibiting both Gram-positive, and Gram-negative organisms (**Table 1**). On the other hand, none of the endophytic isolates showed any ability to inhibit the growth of any of the five fungi tested in this study. From those 18 active strains, all of them inhibited *B. subtilis,* while *E. coli* was not inhibited by only one of the four isolated strains of *N. sphaerica*, *S. aureus,* and *K. pneumoniae* were inhibited by 77.78% (14/18) and 72.22% (13/18) of the active endophytic strains, respectively. On the other hand, *P. aeruginosa* was only inhibited by one strain of *N. sphaerica*.

**Table 1:** Antibacterial activity of endophytic fungi isolated from leaves of *Indigofera suffruticosa*.

	Endophytic fungi	Inhibition diameter zone (mm)				
		*Staphylococcus aureus*	*Bacillus subtilis*	*Escherichia coli*	*Klebsiella pneumoniae*	*Pseudomonas aeruginosa*
ISEF 1	*Colletotrichum gloeosporioides*	26 ± 2^a^	15 ± 0^a^	22 ± 1^a^	22 ± 2^a^	0 ± 0^a^
ISEF 2	*C. gloeosporioides*	16 ± 1.73^b^	24 ± 1^b^	15 ± 1.73^b^	28 ± 2^b^	0 ± 0^a^
ISEF 3	*C. gloeosporioides*	26 ± 1^a^	13 ± 1.73^c^	10 ± 1^c^	26 ± 0^b^	0 ± 0^a^
ISEF 4	*C. gloeosporioides*	22 ± 2^c^	12 ± 0^c^	10 ± 0^c^	26 ± 1^b^	0 ± 0^a^
ISEF 5	*Colletotrichum dematium*	0 ± 0^d^	20 ± 1^d^	11 ± 1.73^c^	24 ± 1^a^	0 ± 0^a^
ISEF 6	*Curvularia pallescens*	0 ± 0^d^	11 ± 0^c^	10 ± 0^c^	0 ± 0^c^	0 ± 0^a^
ISEF 7	*C. pallescens*	0 ± 0^d^	12 ± 1^c^	12 ± 0^c^	0 ± 0^c^	0 ± 0^a^
ISEF 8	*Lasiodiplodia theobromae*	14 ± 0^e^	15 ± 1.73^a,c^	21 ± 1.73^a^	23 ± 1.73^a^	0 ± 0^a^
ISEF 9	*L. theobromae*	32 ± 2^f^	13 ± 1.73^c^	20 ± 1^a^	12 ± 1^d^	0 ± 0^a^
ISEF 10	*Mycelia sterilia*	22 ± 1^c^	22 ± 1^b,d^	14 ± 2.64^b^	26 ± 1^b^	0 ± 0^a^
ISEF 11	*M. sterilia*	18 ± 1^b^	32 ± 1.73^e^	13 ± 1^b^	21 ± 1.73^a^	0 ± 0^a^
ISEF 12	*M. sterilia*	14 ± 1.73^b,e^	15 ± 1.73^a^	10 ± 0^c^	15 ± 1	0 ± 0^a^
ISEF 13	*Nigrospora sphaerica*	16 ± 1.73^b^	14 ± 0^c^	0 ± 0^d^	0 ± 0^c^	0 ± 0^a^
ISEF 14	*N. sphaerica*	12 ± 1^e^	12 ± 1^c^	14 ± 1^b^	0 ± 0^c^	0 ± 0^a^
ISEF 15	*N. sphaerica*	13 ± 1.73^e^	16 ± 0^a^	14 ± 0^b^	12 ± 0^d^	0 ± 0^a^
ISEF 16	*N. sphaerica* (URM-6060)	36 ± 1^f^	28 ± 2^f^	34 ± 1.73^e^	28 ± 1^b^	12 ± 1.73^b^
ISEF 17	*Pestalotiopsis maculans* (URM-6061)	34 ± 1^f^	28 ± 1^f^	26 ± 1^f^	27 ± 1^b^	0 ± 0^a^
ISEF 18	*Phomopsis archeri*	0 ± 0^d^	14 ± 1.15^a,c^	14 ± 1^b^	0 ± 0^c^	0 ± 0^a^

The same superscript letter^(a-f)^ indicates no significant difference (*p* > 0.05) between inhibition diameter zones (IDZ) values from different endophytic fungi against each pathogen (same column).

The strains *N. sphaerica* (URM-6060) and *Pestalotiopsis maculans* (URM-6061) showed the best action, and no significant differences (*p* > 0.05) were observed between their IDZ against most of the tested pathogens (except to *E. coli* and *P. aeruginosa*, which were more efficiently inhibited by the strain URM-6060) These two strains were stored in the URM Culture Collection (WDCM604) and submitted to the other assays.

### Fermentation Assays in Liquid and Semisolid Medium

The antimicrobial activity of the two most active strains (*N. sphaerica* URM-6060 and *P. maculans* URM-6061) was evaluated by liquid and semisolid fermentation assays using different growth media. *N. sphaerica* URM-6060 showed antimicrobial activity against all tested bacteria, but only when it was grown in rice and PDB media. The best activity was observed in PDB medium against all pathogens (*p* < 0.05) with inhibition zones of 36, 32, 30, 28, and 26 mm against *B. subtilis*, *S. aureus*, *E. coli*, *P. aeruginosa,* and *K. pneumoniae*, respectively, (**Table 2**). When cultivated in SAB, the strain URM-6060 only inhibited the growth of *E*. *coli* (20 mm). In semi-solid media, only the methanol extract obtained from rice medium (NsME) showed antimicrobial activity with IDZ ranging from 18 (*B. subtilis*) to 11 mm (*K. pneumoniae*). Interestingly, a strong correlation could be observed between the IDZ values obtained with PDB and rice media (ρ = 0.97). On the other hand, the strain *P. maculans* URM-6061 was not able to produce extracellular antimicrobial compounds in any of the tested media.

**Table 2:** Antimicrobial activity *N. sphaerica* (URM-6060) isolated from leaves of *I. suffruticosa* cultivated in different growth media.

Culture medium	Inhibition diameter zone (mm)				
	*S. aureus*	*B. subtilis*	*E. coli*	*K. pneumoniae*	*P. aeruginosa*
SAB	0 ± 0^a^	0 ± 0^a^	20 ± 1^a^	0 ± 0^a^	0 ± 0^a^
MEB	0 ± 0^a^	0 ± 0^a^	0 ± 0^b^	0 ± 0^a^	0 ± 0^a^
EPM	0 ± 0^a^	0 ± 0^a^	0 ± 0^b^	0 ± 0^a^	0 ± 0^a^
PDB	30 ± 1^b^	36 ± 1^b^	32 ± 2^c^	26 ± 1^b^	28 ± 1^b^
Rice	15 ± 1.73^c^	18 ± 2^c^	16 ± 1^d^	11 ± 1.73^c^	12 ± 1^c^
Corn	0 ± 0^a^	0 ± 0^a^	0 ± 0^b^	0 ± 0^a^	0 ± 0^a^

The same superscript letter^(a-d)^ indicates no significant difference (*p* > 0.05) between IDZ values from strain URM-6060 cultivated in different growth media against each pathogen (same column).

### Minimum Inhibitory and Minimum Bactericidal Concentrations of the NsME and NsEAE Extracts of the Endophytic Fungus *N. sphaerica* (URM-6060)

The NsME (methanolic extract obtained from rice culture medium) and NsEAE (ethyl acetate extract obtained from the filtrate of PDB medium) were subjected to the microdilution test to determine the MIC and MBC, as shown in **Table 3**. NsME was more active against *S. aureus*, *B. subtilis,* and *P.* aeruginosa (MIC of 1.56 mg/mL for all), followed by *E. coli* and *K. pneumoniae* (MIC of 6.25 mg/mL). Furthermore, the MBC values of NsME ranged from 6.25 to 50 mg/mL, and it predominantly showed bacteriostatic actions (MBC/MIC ≥ 4; Pankey and Sabath, 2004; except for *K. pneumoniae*; MBC/MIC ratio of 2).

**Table 3:** Minimum Inhibitory and Minimum Bactericidal Concentrations of extracts of *N. sphaerica* (URM-6060) isolated from leaves of *I. suffruticosa* against human pathogens.

Extract	Concentration (mg/mL)								
	*S. aureus*		*B. subtilis*		*E. coli*		*K. pneumoniae*		*P. aeruginosa*	
	MIC	MBC	MIC	MBC	MIC	MBC	MIC	MBC	MIC	MBC
NsME	1.56	6.25	1.56	12.5	6.25	50	6.25	12.5	1.56	25
NsEAE	0.39	3.12	1.56	6.25	1.56	25	3.12	12.5	1.56	12.5

NsME – methanolic extract obtained from rice culture medium.

NsEAE – ethyl acetate extract obtained from the filtrate of the culture in Potato Dextrose Broth (PDB) medium.

The best antimicrobial action was found with NsEAE as its MIC values ranged from 0.39 to 3.12 mg/mL and MBC values from 3.12 to 50 mg/mL, respectively. In fact the strongest activity was found against *S. aureus* (MIC of 0.39 mg/mL and MCB of 3.12 mg/mL), followed by *B. subtilis* (MIC of 1.56 mg/mL and MCB of 6.25 mg/mL) and *P. aeruginosa* (MIC of 1.56 mg/mL and MCB of 12.5 mg/mL), *E. coli* (MIC of 1.56 mg/mL and MCB of 25 mg/mL), and *K. pneumoniae* (MIC of 3.12 mg/mL and MCB of 12.5 mg/mL). This extract also showed bacteriostatic actions (MBC/MIC ≥ 4).

### Phytochemical Screening of the NsME and NsEAE Extracts of the Endophytic Fungus *N. sphaerica* (URM-6060)

Phytochemical analysis revealed the presence of terpenoids, steroids, hydrolyzable tannins, alkaloids, and cinnamic derivatives (**Table 4**). Both extracts showed the same chemical constitution, except for the presence of anthracene derivatives in NsEAE. All these classes of compounds have been reported as antimicrobial agents.

**Table 4:** Phytochemical analysis of the extracts from *N. sphaerica* (URM-6060) isolated from leaves of *I. suffruticosa*.

Extract	Compound								
	Steroid	Saponin	Alkaloid	Flavonoid	Tannin	Sugar	Terpenoids	Cinnamic derivatives	Anthracene derivatives
NsME	+	-	+	-	+	+	+	+	-
NsEAE	+	-	+	-	+	+	+	+	+

NsME – methanolic extract obtained from rice culture medium.

NsEAE – ethyl acetate extract obtained from the filtrate of the culture in PDB medium.

## Discussion

One of the most important properties of endophytic microorganisms, especially fungi, is linked to their metabolic potential to produce a large variety of bioactive molecules that can protect the plant against pathogens (Tan and Zou, 2001; Strobel, 2003). For example, natural compounds synthesized by endophytic fungi have been reported as inhibitors of a wide variety of animal and plant pathogens (Wiyakrutta et al., 2004; Gunatilaka, 2006; Zhao et al., 2011b). The isolation and identification of endophytic mycobiota is necessary, since the medicinal properties of a plant can be a consequence of the capacity of its endophytic microorganisms to produce biologically active secondary metabolites (Kaul et al., 2012; Kusari et al., 2013). This was the case in the classic example of taxol, an anticancer agent produced by *Taxus brevifolia* Nutt., and its endophyte *Taxomyces andreanae* (Stierle et al., 1993).

In the present work 65 endophytic strains were isolated from the medicinal plant *I. suffruticosa* and the antimicrobial activity of them was evaluated. A total of 18 (33.6%) strains showed antibacterial activity, most of them (17/18; 94.44%) with wide spectrum. The percentage of endophytic fungi isolated from leaves of *I. suffruticosa* showed that antimicrobial activity was comparable and even exceeding some results reported by other authors in similar studies, revealing the enormous capacity of production of bioactive compounds with antimicrobial potential by these microorganisms. For example, only 8.3% of the strains isolated from *Dracaena cambodiana* and *Aquilaria sinensis* showed antimicrobial activity (Gong and Guo, 2009), whereas 27.6% of strains isolated from *Camptotheca acuminata* displayed antimicrobial activity against some pathogens (Lin et al., 2007).

The two most active strains (*N. sphaerica* URM-6060 and *P. maculans* URM-6061) were further examined using liquid and semi-solid fermentation assays. Only *N. sphaerica* URM-6060 showed antibacterial activity (only in PDB, SAB, and rice media), and the best results were found using PDB medium. Similar results were observed by Siqueira et al. (2011) in a study of the antimicrobial activity of endophytic fungi from *Lippia sidoides* Cham., where 16 out of 203 endophytic isolates showed antimicrobial activity in an assay on solid medium, and of the 16 endophytic fungi which were submitted to the fermentation assay, 10 displayed antimicrobial activity. The production of a bioactive compound by an endophyte can be stimulated in the host plant or by a host plant extract. When grown *in vitro*, an endophyte may continue to produce bioactive material, or this may cease after a certain time. Research is needed to discover what factors could encourage endophytes to continue synthesizing compounds *in vitro* (Owen and Hundley, 2004).

Endophytic fungi from the genus *Nigrospora* have been reported as rich sources of bioactive secondary metabolites with applications in various fields. Such secondary metabolites include herbicidal phomalactone (Kim et al., 2001), phytotoxic and antibacterial nigrosporins (Tanaka et al., 1997), phytotoxic lactones (Fukushima et al., 1998), and activity against plant pathogenic fungi (Zhao et al., 2012). In this work the best antimicrobial action was found with NsEAE, this extract was most active against *S. aureus*. This extract is composed of terpenoids, steroids, hydrolyzable tannins, alkaloids, cinnamic derivatives, and anthracene derivatives, all of them reported to be antimicrobial agents (Funatogawa et al., 2004; Yu et al., 2010; Du et al., 2012; Sova, 2012; Mousa and Raizada, 2013).

Specifically, the production of terpenoids by endophytic fungi and their biological activities were reported in a recent review (Souza et al., 2011). The steroid ergosta-7,9 (14), 22-triene-3β-ol, produced by the endophytic fungus *N. sphaerica* isolated from leaves of *Vinca rosea*, showed antifungal activity against *Cryptococcus neoformans* with an IC50 value of 14.81 μg/mL (Metwaly et al., 2014). The incubation temperature, medium composition, and degree of aeration affect the amount and kinds of compounds that are produced by an endophytic fungus (Strobel et al., 2004). The culture medium, agitation, and temperature can increase or reduce the production of the bioactive compounds by fungi. For this reason, further tests are needed to evaluate the biological activity of the strains that showed inhibition on solid media but did not produce bioactive compounds during the fermentation assay. The production of a bioactive compound by an endophyte can be stimulated in the host plant or by a host plant extract. Other explanations for this may be the presence of some inhibitory compound in the extract, the range of concentration of extract tested and the pathogenic fungi selected for the test (Pawle and Singh, 2014).

Furthermore, our observations indicate that endophytic fungi from leaves of *I. suffruticosa* have pharmaceutical potential as they produce antimicrobial compounds, and that the medicinal properties of this plant may be a consequence of the capacity of its endophytic microorganisms to produce biologically active secondary metabolites. Further studies are now needed to identify the active compounds produced in order to discover new drugs with antibacterial activity.

## Author Contributions

Conceived and designed the experiments: IPS, VLML, and MSC. Performed the experiments: IPS and MSC. Analyzed the data: VLML, MSC, LCNA, and JMA. Contributed reagents/materials/analysis tools: VLML, MSC, and JMA. Wrote and enriched the literature: IPS, LCNA, VLML, and MVS.

## Conflict of Interest Statement

The authors declare that the research was conducted in the absence of any commercial or financial relationships that could be construed as a potential conflict of interest.
